# Identification of Lipophagy-Related Gene Signature for Diagnosis and Risk Prediction of Alzheimer’s Disease

**DOI:** 10.3390/biomedicines13020362

**Published:** 2025-02-05

**Authors:** Hongxiu Guo, Siyi Zheng, Shangqi Sun, Xueying Shi, Xiufeng Wang, Yang Yang, Rong Ma, Gang Li

**Affiliations:** 1 Department of Neurology, Union Hospital, Tongji Medical College, Huazhong University of Science and Technology, Wuhan 430022, China; ghx2638390443@163.com (H.G.);; 2 Department of General Medicine, Binzhou Medical University Hospital, Binzhou 256603, China; 3 Department of Pharmacology, School of Basic Medicine, Tongji Medical College, Huazhong University of Science and Technology, Wuhan 430022, China

**Keywords:** Alzheimer’s disease, autophagy, biomarkers, lipid metabolism, prediction

## Abstract

**Background:** Recent research indicates that lipid metabolism and autophagy play crucial roles in the development of Alzheimer’s disease (AD). Investigating the relationship between AD diagnosis and gene expression related to lipid metabolism, autophagy, and lipophagy may improve early diagnosis and the identification of therapeutic targets. **Methods:** Transcription datasets from AD patients were obtained from the Gene Expression Omnibus (GEO). Genes associated with lipid metabolism, autophagy, and lipophagy were sourced from the Gene Set Enrichment Analysis (GSEA) database and the Human Autophagy Database (HADb). Lipophagy-related hub genes were identified using a combination of Limma analysis, weighted gene co-expression network analysis (WGCNA), and machine learning techniques. Based on these hub genes, we developed an AD risk prediction nomogram and validated its diagnostic accuracy using three external validation datasets. Additionally, the expression levels of the hub genes were assessed through quantitative reverse transcription polymerase chain reaction (qRT-PCR). **Results:** Our analysis identified three hub genes—*ACBD5*, *GABARAPL1*, and *HSPA8*—as being associated with AD progression. The nomogram constructed from these hub genes achieved an area under the curve (AUC) value of 0.894 for AD risk prediction, with all validation sets yielding AUC values greater than 0.8, indicating excellent diagnostic efficacy. qRT-PCR results further corroborated the associations between these hub genes and AD development. **Conclusions:** This study identified and validated three lipophagy-related hub genes and developed a reliable diagnostic model, offering insights into the pathology of AD and facilitating the diagnosis of AD patients.

## 1. Introduction

Alzheimer’s disease (AD) is the leading cause of dementia, affecting approximately 50 million individuals worldwide, with projections indicating a threefold increase by 2050 [1]. Characterized by the accumulation of amyloid-β plaques and the propagation of pathological tau protein, AD leads to progressive cognitive impairment and ultimately results in dependency on caregivers [2]. Currently, there is no effective treatment available, and individuals diagnosed with Alzheimer’s disease dementia have a median survival time of six years [3]. According to data from official death certificates, 121,499 deaths were attributed to Alzheimer’s disease in 2019, making it the fifth leading cause of death among individuals aged 65 and older [4]. While the etiology of AD remains unclear, evidence suggests that 60–80% of the risk for developing the disease is heritable [5]. Therefore, identifying causative or protective genes is crucial for the development of potential diagnostic and therapeutic interventions for Alzheimer’s disease.

There are some molecules that play important roles in the pathogenesis of AD. Lipids, one of these, constitute 50–60% of the dry weight of the brain, playing important roles in energy storage, membrane formation, neurogenesis, cellular trafficking, and signal transduction [6]. Recent research has reported the impact of lipids on processes of AD [7,8,9]. The *ApoE* gene is a crucial factor in lipid transport and is recognized as an important genetic risk factor for AD [10]. Patients with the *APOE4/4* genotype have been reported to exhibit a high abundance of lipid-droplet-accumulating microglia (LDAM), which indicate a dysfunctional and pro-inflammatory state in the aging brain. Furthermore, conditioned media from LDAM have been shown to induce tau phosphorylation and neurotoxicity in an *APOE*-dependent manner [11]. Lipid rafts (LR), which are rich in various lipid components such as gangliosides, phospholipids, cholesterol, and sphingolipids, play a significant role as membrane microdomains in the processing of amyloid precursor protein (APP) [12]. Disruption of the metabolism of these lipids can impair LR function, leading to enhanced generation and aggregation of amyloid-beta (Aβ), thereby exacerbating the neuropathology associated with AD.

Lipophagy is a selective form of autophagy that facilitates the degradation of lipid components within lipid droplets (LDs) through the activation of autophagy-related molecules. The catabolized products from this process are utilized in the β-oxidation pathway, providing energy and contributing to the synthesis of other lipids necessary for maintaining biological functions [13]. In patients with Alzheimer’s disease (AD), autophagic dysfunction and the accumulation of LDs in the hippocampus have been reported [14,15]. Evidence suggests that impaired autophagy can disrupt lipid homeostasis, exacerbate tau pathology and its propagation, and lead to cognitive impairment [16]. However, the precise mechanisms underlying lipophagy, as well as the key genes and molecules involved in this process, remain unclear and require further investigation.

With advancements in bioinformatics research techniques, researchers can identify significant genes associated with disease etiology, thereby facilitating potential diagnostic and management strategies. Several bioinformatics-based studies have identified molecules related to Alzheimer’s disease and explored the underlying mechanisms [17,18,19]. In this study, we aimed to characterize the molecular features of lipophagy-related genes (Lipos) in Alzheimer’s disease and develop a predictive signature for Alzheimer’s risk using bioinformatics and machine learning tools. Furthermore, we validated the hub genes and the predictive signature using three additional external datasets.

## 2. Materials and Methods

### 2.1. Data Acquisition

AD-related datasets were retrieved from the GEO database (https://www.ncbi.nlm.nih.gov/geo/, accessed on 18 December 2023). GSE5281 (GPL570) contained 74 cases of AD and 87 controls were used as a training cohort, GSE28146 (GPL570, 22 AD cases and 8 controls), GSE122063 (GPL16699, 56 AD cases and 44 controls), and GSE132903 (GPL10558, 97 AD cases and 98 controls) served as validation cohorts. A total of 1190 lipid metabolism-related genes and 9 lipophagy-related genes were selected from the GSEA database (https://www.gsea-msigdb.org/gsea/login.jsp, accessed on 2 January 2024), while 661 autophagy-related genes were obtained from GSEA database and HADb (http://www.autophagy.lu/index.html, accessed on 2 January 2024). The pertinent details are displayed in Table S1 in the Supplementary Materials.

### 2.2. Identification of Differentially Expressed Genes (DEGs) and Weighted Gene Co-Expression Network Analysis (WGCNA)

The adjusted *p*-value < 0.05 and |log2(FoldChange)| > 2 were defined as screening criteria to evaluate DEGs between AD and healthy controls in the training cohort utilizing “Limma” R package(version 4.4.1). The R packages “ggplot2” and “pheatmap” were used to plot the volcano plot and heatmap of DEGs, respectively. Weighted gene co-expression network analysis (WGCNA) [20] with the function of gene–trait association analysis was performed to identify key modules of highly correlated genes in AD patients. Specifically, the sample data is first preprocessed to eliminate outliers and was used to construct a correlation matrix with the help of the R package “WGCNA”. We then selected the optimal soft threshold to transform the correlation matrix into an adjacency matrix. This was followed by generating a topological overlap matrix (TOM). Finally, we employed average linkage hierarchical clustering, using the TOM-based phase dissimilarity metric, to group genes with similar expression patterns into distinct gene modules. The four modules with the strongest relevance to AD occurrence were identified as key modules. The genes in key modules and DEGs were intersected as WGCNA-DEGs for further analysis. The WGCNA-DEGs were intersected with lipid metabolism and autophagy (LA) or lipophagy (Lipo)-related genes to obtain LA/Lipo-related DEGs for subsequent study.

### 2.3. Gene Ontology (GO) and Kyoto Encyclopedia of Genes and Genomes (KEGG) Functional Enrichment Analysis

To determine the roles of lipid metabolism-related DEGs and autophagy-related DEGs, the “clusterProfiler” R package was used to perform GO and KEGG functional enrichment analyses [21] to assess molecular functions (MFs), biological processes (BPs), cellular components (CCs), and potential signaling pathways of these genes. The findings of GO and KEGG were plotted with the “ggplot” and “GOplot” packages of the R program, respectively.

### 2.4. Gene Expression Patterns and Protein–Protein Interaction Analyses of LA/Lipo-Related DEGs

The expression patterns of LA/Lipo-related DEGs were examined. Initially, the LA/Lipo-related DEGs were identified by analyzing the interactions between lipid metabolism-related DEGs and autophagy-related DEGs, plus one lipophagy-related DEG. The chromosomal locations of the LA/Lipo-related DEGs were then determined and visualized using the RCircos package. Subsequently, the correlation between the expression levels of the LA/Lipo-related DEGs was validated using the corrplot function from the R package corrplot. Additionally, an online website called String (https://www.string-db.org/) and Cytoscape software (version 3.7.1) was utilized to perform a protein–protein interaction (PPI) enrichment analysis. Interactions with a combined score greater than 0.4 were considered statistically significant. The gene interactions were depicted as a network, where individual genes were represented as nodes and the connections represented the entire networks. Additionally, we assessed the relationship between immune cell infiltration and LA/Lipo-related DEGs and depicted the correlation heatmap using the “GSVA” R package.

### 2.5. Identification of LA/Lipo-Related Hub Genes Based on Machine Learning Algorithms

Three machine learning algorithms, including least absolute shrinkage and selection operator (LASSO) [22], random forest (RF) [23], and support vector machine (SVM) [24] were employed to identify hub genes from the differentially expressed lipophagy genes. The R packages “glmnet”, “e1071”, “caret”, “sigFeature”, and “tidyverse” were utilized. LASSO logistic regression selected variables based on their minimal likelihood of classification errors. RF employed an ensemble machine learning technique using independent decision trees for regression or clustering prediction. SVM, on the other hand, created a hyperplane in the feature space to separate negative and positive instances with the maximum margin. The optimal lambda value for LASSO regression was determined, and the performance of RF was assessed through 10-fold cross-validation, while the performances of SVM were evaluated through 5-fold cross-validation. The genes identified as overlapping by all three algorithms were selected as LA/Lipo-related hub genes.

### 2.6. Construction and Validation of a Nomogram Model for AD Risk Prediction

Using the “rms” and “gglot2” packages, a multivariate logistic regression analysis was conducted, and a model of nomogram was developed for the diagnosis of AD based on hub genes associated with lipophagy. The “total points” in the nomogram represented the cumulative score assigned to the predictors, with each predictor having an associated point value. The receiver operating characteristic (ROC) curves were generated using the “ROC” R package to assess the diagnostic performance of the nomogram, while calculating the area under the curve (AUC). Meanwhile, a calibration curve was drawn using the “pacman” package to verify the accuracy of the nomogram, and a decision curve analysis (DCA) was performed using the “limma” package to evaluate the clinical practical value of the model. Finally, the ROC analysis was performed in three validation cohorts to further evaluate the predictive performance of the prediction model.

### 2.7. Consensus Cluster Analysis

Consensus clustering [25] is an unsupervised hierarchical clustering, which was employed to identify molecular subtypes based on the expression of LA/Lipo-related hub genes in the training set. The maximum number of categories to be assessed is five, and each k is iterated 50 times. The clustering distance chosen is the Euclidean distance. The number of clusters was determined using the consensus clustering algorithm implemented in the “ConsensuClusterPlus” R package.

### 2.8. Immune Cell Infiltration and Gene Set Enrichment Analysis (GSEA) 

The immune activity of 161 samples in the training cohort were assessed using Cell-type Identification by Estimating Relative Subsets of RNA Transcripts (CIBERSORT) and the ssGSEA algorithm [26] from the “GSVA” R package. The relative abundance of immune cells was quantified, and the differences in immune infiltration between AD and control groups were examined. Additionally, the enrichment scores between two sub-clusters were compared, and the “ggpubr” R package was used to visualize the results. A single-gene GSEA [27] analysis was performed to investigate the possible signaling pathways of three hub genes. We obtained the “c2.cp.kegg.v7.0.entrez.gmt” file from the MsigDB database to conduct the analysis. A statistically significant difference in expression between the high and low expression groups was indicated by an adjusted *p*-value less than 0.05.

### 2.9. TF/miRNA-Gene Regulatory Networks and Drug–Gene Interaction Predication

To explore the molecules responsible for regulating gene expression, we used NetworkAnalyst (https://www.networkanalyst.ca/, accessed on 28 March 2024) to obtain potential transcription factors (TFs) and miRNAs and presented the interaction networks utilizing Cytoscape software. We searched the DSigDB database for drugs or small organic compounds for LA/Lipo-related hub genes, which were considered potential pharmaceutical targets for AD treatment. We selected the drugs with an adjusted *p*-value < 0.05 as latent therapeutic drugs for AD.

### 2.10. Animals

P301S transgenic mice [B6; C3-Tg (Prnp-MAPT/P301S) PS19Vle/J], a well-established model of Alzheimer’s disease (AD), were obtained from the Jackson Laboratory (Bar Harbor, ME, USA). For this study, male P301S mice (n = 6) served as the AD models, while age-matched male wild-type littermates (n = 6) were included as the control group. All animals were housed under standard laboratory conditions, which included a 12-h light/dark cycle, controlled temperature, and unrestricted access to food and water. At nine months of age, cortical regions of the mice were surgically isolated on ice and subsequently stored at −80 °C for biochemical analysis. The animal experiments were reviewed and approved by the Ethics Committee of Tongji Medical College, Huazhong University of Science and Technology.

### 2.11. Quantitative Reverse-Transcription Polymerase Chain Reaction (qRT-PCR)

Fresh brain tissue was homogenized with 500 μL of Buffer RL for every 10–20 mg of tissue using an electric homogenizer until no visible tissue mass remained. Total RNA was extracted from the homogenates and reverse transcribed into complementary DNA (cDNA) using an RNA extraction and reverse transcription kit (Vazyme, Nanjing, China; RC323). Quantitative real-time PCR (qRT-PCR) experiments were subsequently performed and analyzed using a StepOnePlus real-time PCR system (Takara, RR096A). The mRNA expression levels of the target genes were normalized to the reference gene β-actin, and relative expression levels were calculated using the 2^−ΔΔCt^ method. The primer sequences utilized in the qRT-PCR assays are provided in Supplementary Table S2.

### 2.12. Statistical Analysis

All statistical analyses were conducted using R 4.3.1 and GraphPad Prism 9. The diagnostic value of the predictive model was evaluated using univariate and multivariate logistic regression analyses. Comparisons between groups were performed using unpaired Student’s *t*-tests. All statistical tests were two-sided, and a *p* value < 0.05 was deemed to indicate a statistically significant difference.

## 3. Results

### 3.1. Identification of DEGs and Construction of Weighted Gene Co-Expression Networks

**Figure 1:**
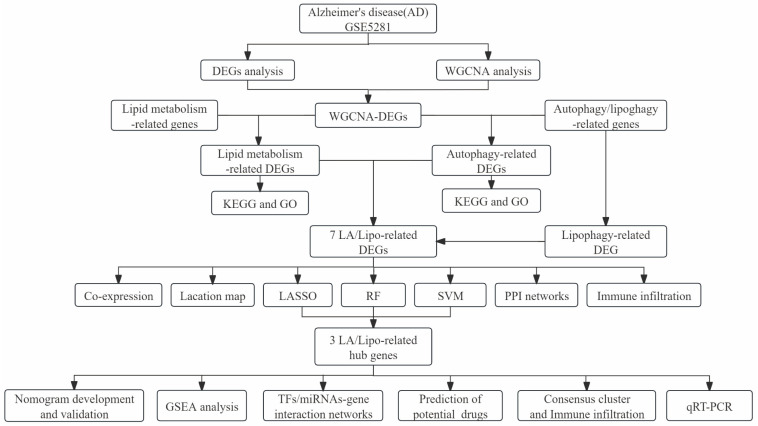
The flowchart of the study.

**Figure 2:**
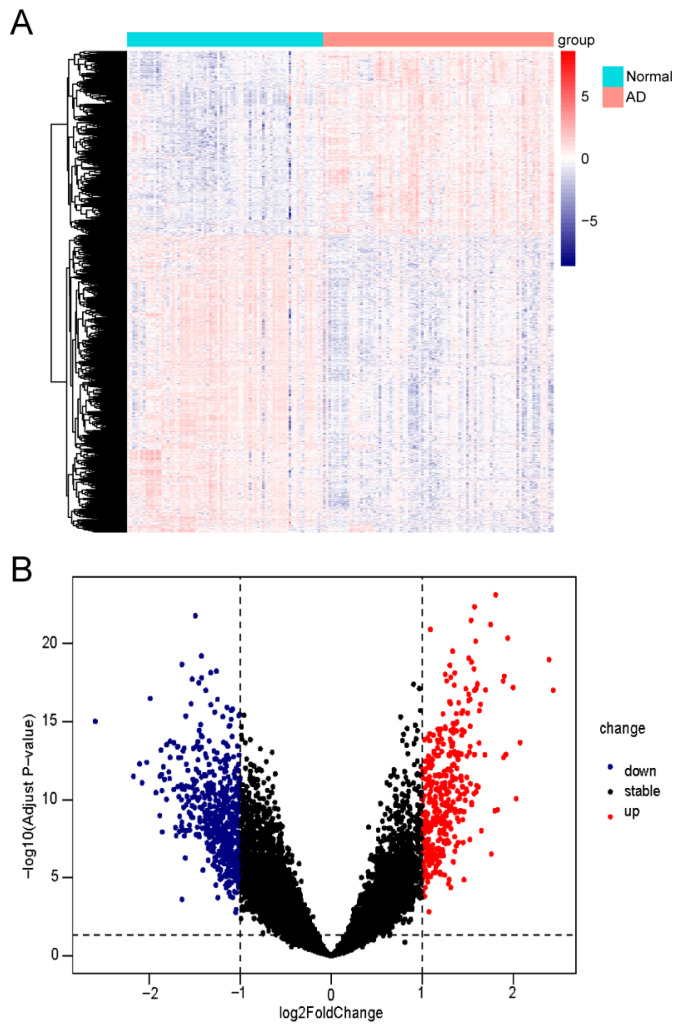
Identification of AD-related DEGs. (**A**) Heat map of differentially expressed genes between AD samples and control samples. (**B**) Volcano plot of differentially expressed genes between AD samples and control samples (|log2(FoldChange)| = 2 were defined as screening criteria as the horizontal dashed line presented).

The detailed study flowchart is shown in Figure 1. A total of 20824 genes in 161 samples were acquired from GSE5281 and normalized with log2 transformation before differential analysis. Using the cutoff values of |log2 fold change (FC)| greater than 1 and an adjusted *p*-value less than 0.05, a total of 961 DEGs were detected, including 367 upregulated and 594 downregulated genes. The volcano plot and heat map of DEGs are presented in Figure 2A,B.

**Figure 3:**
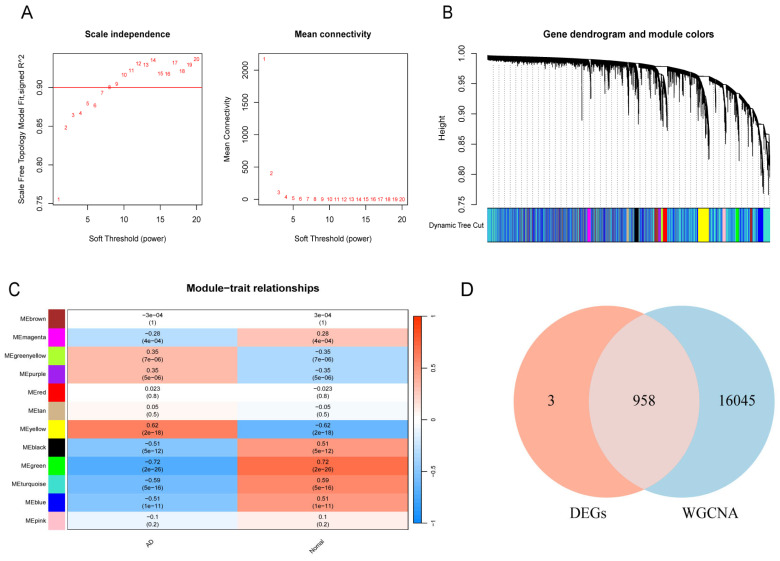
Construction of weighted gene co-expression networks (WGCNA). (**A**) Choosing the best soft-threshold power. (**B**) Gene dendrogram and module colors. (**C**) The 12 modules associated with AD revealed by the WGCNA (4e-04 means 4 × 10^−4^, ect.). (**D**) Venn of DEGs and WGCNA.

The soft threshold power of 3 was selected with scale-free R2 > 0.85, and the mean connectivity decreases substantially (Figure 3A). Gene modules were partitioned using a dynamic cutting method, and subsequently, all modules were clustered to obtain the final modules using the “mergeCloseModules” function (Figure 3B). The “yellow”, “green”, “black”, “blue”, and “turquoise” modules consisted of 17003 AD-related genes and were selected as the top correlated modules with the *p*-values of Pearson correlation analysis showing significant statistical difference (Figure 3C). By overlapping the DEGs and the genes belonging to the top modules identified by the WGCNA, a set of 958 WGCNA-DEGs were generated for further analysis (Figure 3D).

### 3.2. GO and KEGG Functional Enrichment Analysis

**Figure 4:**
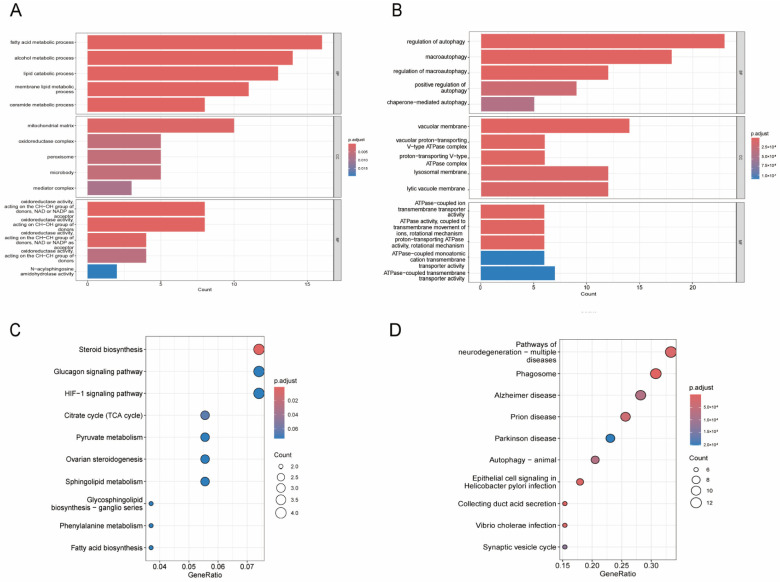
Enrichment analyses of lipid metabolism-related DEGs and autophagy-related DEGs. (**A**) The top 5 most significantly enriched GO terms of lipid metabolism-related DEGs. (**B**) The top 5 most significantly enriched GO terms of autophagy-related DEGs. (**C**) The top 10 most significantly enriched KEGG pathways of lipid metabolism-related DEGs. (**D**) The top 10 most significantly enriched KEGG pathways of autophagy-related DEGs.

A total of 90 autophagy-related DEGs and 136 lipid metabolism-related DEGs were obtained through intersecting the autophagy/lipid metabolism-related genes and WGCNA-DEGs. We conducted GO and KEGG functional enrichment analysis to investigate the possible biological processes and pathways of targeted DEGs. The findings of analyses indicated that lipid metabolism-related DEGs were mainly involved in biological processes, including the fatty acid metabolic process, alcohol metabolic process, and lipid catabolic process, and in the signaling pathways, including steroid biosynthesis, glucagon, and HIF-1 signaling pathways (Figure 4A,B). The most significant biological processes related to autophagy-related DEGs were regulation of autophagy and macro-autophagy, while signaling pathways were identified as having close relationships with neurodegeneration relating to multiple diseases, phagosomes, and Alzheimer’s disease (Figure 4C,D).

### 3.3. Gene Expression Patterns and PPI Network of LA/Lipo-Related DEGs

**Figure 5:**
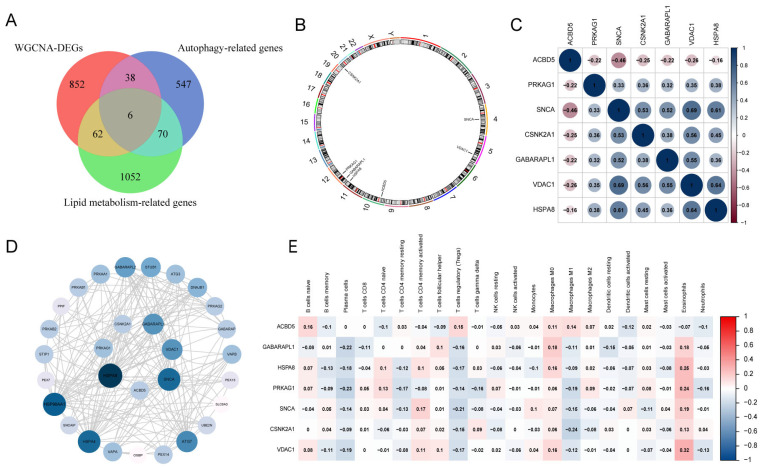
Gene expression patterns, PPI network, and immune cell infiltration of LA/Lipo-related DEGs. (**A**) The overlap of genes among WGCNA-DEGs, lipid metabolism-related genes, and autophagy-related genes. (**B**) The location of the LA/Lipo-related DEGs on chromosomes. (**C**) Correlation matrix of LA/Lipo-related DEGs. (**D**) PPI network of LA/Lipo-related DEGs and its interacting proteins. (**E**) Immune cell infiltration analysis of LA/Lipo-related DEGs. PPI, protein–protein interaction; DEGs, differentially expressed genes.

The interaction of DEGs with autophagy and lipid metabolism-related genes resulted in six LA-related DEGs (Figure 5A). One lipophagy-related DEG was added to form seven LA/Lipo-related DEGs for subsequent analysis. The locations of these DEGs on chromosomes were *ACBD5* (chr10), *GABARAPL1* (chr12), *HSPA8* (chr11), *CSNK2A1* (chr20), *PRKAG1* (chr12), *SNCA* (chr4), and *VDAC1*(chr5) (Figure 5B). Correlations among these LA/Lipo-related DEGs are presented in the gene relationship network diagram (Figure 5C). We constructed a PPI network comprising 23 other genes including *ATG3, ATG7, DNAJB1, GABARAP, GABARAPL2, HSP90AA1, HSPA4, OSBP, PEX13, PEX14, PEX7, PPIF, PRKAA1, PRKAB1, PRKAB2, PRKAG2, SLC6A3, SNCAIP, STIP1, STUB1, UBE2N, VAPA*, and *VAPB* (Figure 5D). Additionally, the correlation heatmap of immune cell infiltration showed most of these LA/Lipo-related DEGs have low expression levels in regulatory T cells (Tregs) and M1 macrophages, plasma cells, and they have high levels in M0 macrophages and eosinophils (Figure 5E).

### 3.4. Identification of LA/Lipo-Related Hub Genes via Machine Learning Algorithm

**Figure 6:**
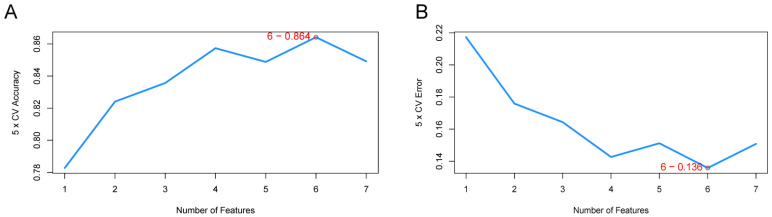

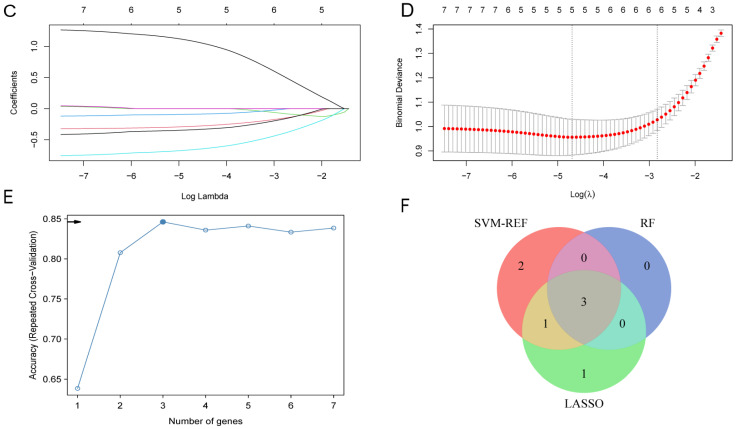
Identification of LA/Lipo-related hub genes for AD. (**A,B**) The plot of feature screening via the SVM-RFE arithmetic. (**C**,**D**) Tuning feature identification in the LASSO model. (**E**) The plot of accuracy for RF selection. (**F**) Venn graph displaying 3 genes shared by SVM-RFE, RF, and LASSO algorithms.

The RF, SVM, and LASSO were conducted to identify hub genes associated with the diagnosis of AD. The SVM-RFE model identified six features as hub genes with the highest accuracy and the minimum classification error (Figure 6A,B), while the RF classifier selected three genes, as it has the highest accuracy (Figure 6E). In LASSO regression, five LA/Lipo-related DEGs were recognized as candidate key genes based on the optimal lambda value (Figure 6C,D). Finally, three common genes including *ACBD5*, *GABARAPL1*, and *HSPA8* selected by the above three algorithms were confirmed as hub genes (Figure 6F).

### 3.5. Performance of LA/Lipo-Related Hub Genes

**Figure 7:**
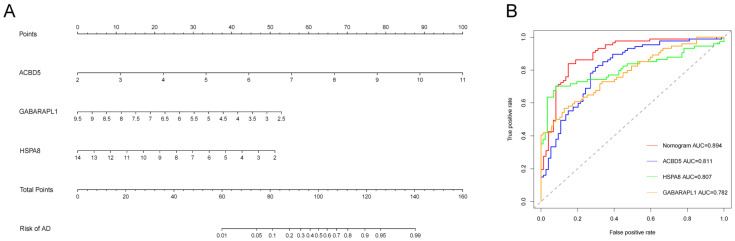

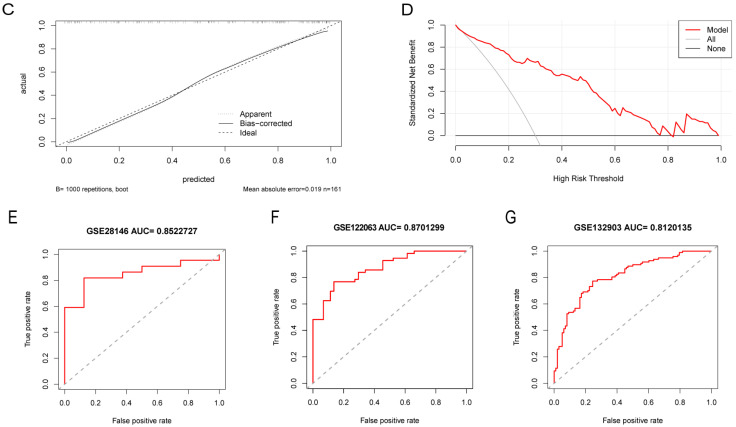
The performance of LA/Lipo-related hub genes and nomogram in training and validation datasets. (**A**) The nomogram model developed combing three hub genes in the GSE5281 training dataset. (**B**) ROC analyses of the nomogram and three hub genes in the training set (The dotted line means AUC = 0.5). (**C**) The calibration curve of the nomogram in the training set. (**D**) The DCA of the nomogram in the training set. (**E**–**G**) ROC curves of the nomogram in the GSE28146, GSE122063, and GSE132903 dataset, respectively.

A nomogram model was developed using three hub genes associated with lipophagy to assess the risk of developing AD (Figure 7A). ROC curve analyses indicated that the nomogram had an AUC value of 0.894 for AD risk prediction, while the AUC values for each hub gene achieved 0.75, demonstrating their excellent diagnostic performance (Figure 7B). Calibration curve analysis of the nomogram exhibited good agreement between the predicted probabilities and observed outcomes (Figure 7C). Decision curve analysis (DCA) further confirmed the clinical utility of the predictive model for decision making (Figure 7D). Additionally, three independent validation datasets, GSE28146, GSE122063, and GSE132903, were utilized to assess the performance of this model using receiver operating characteristic (ROC) curves. The analysis found that the model performed well in the validation cohorts, with area under the curve (AUC) values of 0.852, 0.870, and 0.812, respectively (Figure 7E–G).

### 3.6. Identification LA/Lipo-Related Sub-Clusters and Differences in the Immune Cell Infiltration Between Sub-Clusters

**Figure 8:**
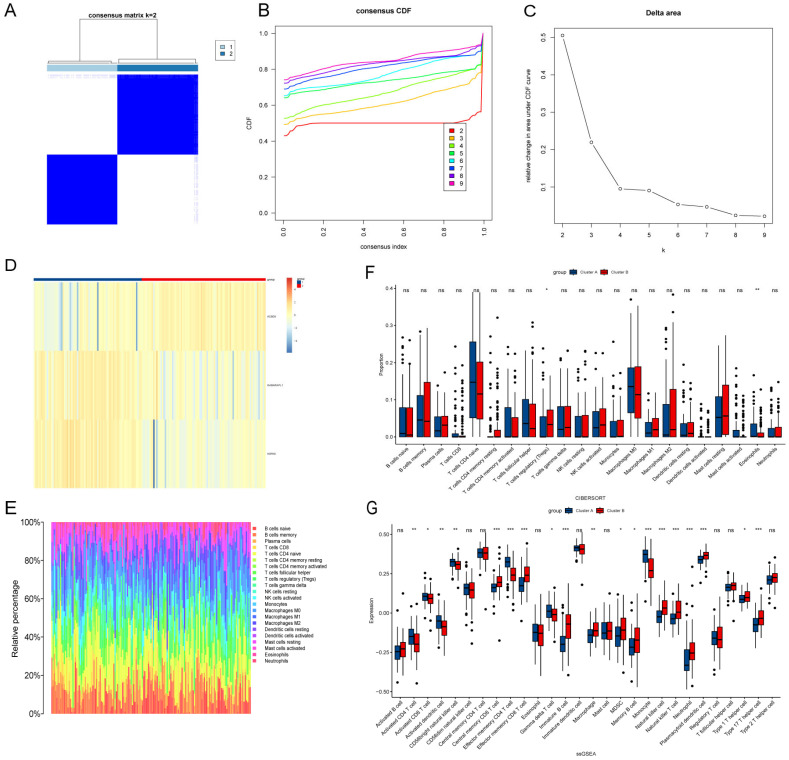
Consensus clustering in AD patients and immune infiltration analysis between clusters. (**A**) Consensus clustering matrix when k = 2. (**B**) Consensus CDF delta area curves when k = 2–9. (**C**) Relative alterations in the area under CDF curve. (**D**) Heat map of three hub genes between cluster A and cluster B. (**E**) The proportion of 28 immunocytes in all samples visualized from the bar plot. (**F**) Comparison of immune cell infiltration levels calculated according to CIBERSORT analysis between the two clusters. (**G**) Comparison of immune cell infiltration levels calculated according to ssGESA analysis between the two clusters. * *p* < 0.05; ** *p* < 0.01; *** *p* < 0.001; ns: *p* > 0.05.

Based on the selected three hub genes, 161 individuals were grouped using a consensus clustering algorithm. The k value of two (k = 2) was selected as the optimal number of clusters, as it provided the best performance and stability for each group (Figure 8A). At k = 2, the relatively small changes in CDF values indicate a high consistency index, reflecting a stable data distribution (Figure 8B). Additionally, the maximized delta area value suggests improved clustering separation (Figure 8C). Under these conditions, *ACBD5* shows increased expression in cluster A, while *GABARAPL1* and *HSPA8* exhibit higher expression levels in cluster B (Figure 8D). Furthermore, we employed the CIBERSORT and ssGSEA algorithms to assess the immune infiltration levels of the two clusters, with the proportions of 28 immune cell types visualized in Figure 8E. Overall, the infiltration proportion of B cells in Alzheimer’s disease (AD) is relatively low, whereas the proportions of neutrophils, mast cells, and natural killer cells are higher. In the Cibersort algorithm, the infiltration levels of regulatory T cells (Tregs) in cluster B were higher than that in A, while cluster A showed higher infiltration levels of eosinophils than B (Figure 8F). In the ssGSEA algorithm, cluster A had higher infiltration levels of activated CD8 T cell, activated CD4 T cell, activated dendritic cell, CD56bright natural killer cell, effector memory CD4 T cell, gamma delta T cell, and monocyte, while higher infiltration levels of central memory CD8 T cell, effector memory, CD8 T cell, immature B cell, macrophage, memory B cell, MDSC, natural killer T cell, natural killer cell, plasmacytoid dendritic cell, neutrophil, type 1/17 T helper cell presented in cluster B (Figure 8G).

### 3.7. GSEA Analysis

**Figure 9:**
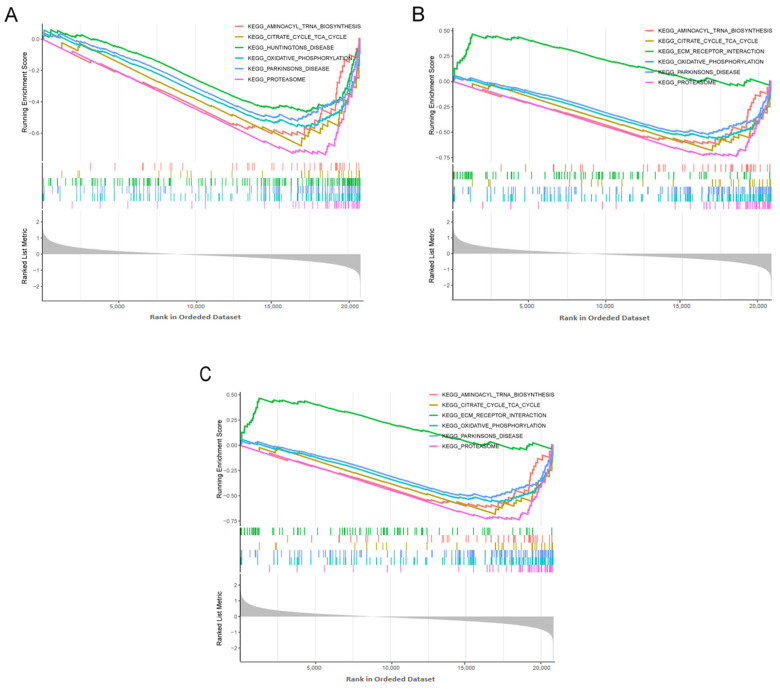
Signaling pathways of hub genes identified using gene set enrichment analysis: (**A**) *ACBD5*, (**B**) *GABARPL1*, (**C**) *HSPA8*.

We identified top relevant signaling pathways associated with the three candidate biomarkers using the GSEA algorithm. According to the GSEA results, the *ACBD5* (Figure 9A), *GABARAPL1* (Figure 9B), and *HSPA8* (Figure 9C) were involved in five pathways, including proteasome, oxidative phosphorylation, citrate cycle TCA cycle, Parkinsons disease, and aminoacyl tRNA biosynthesis. Additionally, the decreased *ACBD5* expression level was associated with Huntington’s disease, while high *GABARAPL1* and *HSPA8* expression levels were concentrated in ECM receptor interaction.

### 3.8. Construction of Regulatory Network and Predication of Drug–Gene Interaction

**Figure 10:**
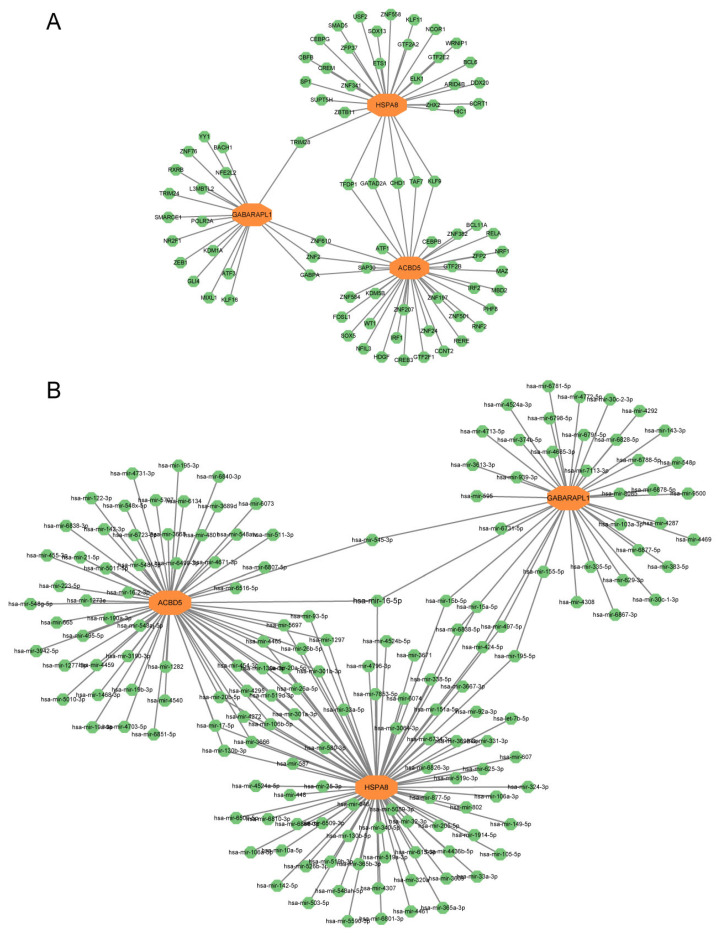
Regulatory network. (**A**) Interaction network of TFs and the hub genes. (**B**) Interaction network of miRNAs and hub genes. TF, transcription factors; miRNA, microRNA.

We identified a total of 80 TFs using the JASPAR database. Among these TFs, nine (TRIM28, GABPA, ZNF2, ZNF610, KLF9, GATAD2A, CHD1, and TAF7) had a degree of 2 or higher (Figure 10A). Additionally, using the TarBase database, we predicted 164 potential miRNAs and identified 21 miRNAs of degree ≥ 2 (Figure 10B). Furthermore, we screened six potential therapeutic agents including 1,4-chrysenequinone, thiostrepton, parthenolide, 15-delta prostaglandin J2, puromycin, and menadione in the DSigDB database, employing an adjusted *p*-value cutoff of <0.05 (Table 1).

### 3.9. Validation of Hub Genes Expression

**Figure 11:**
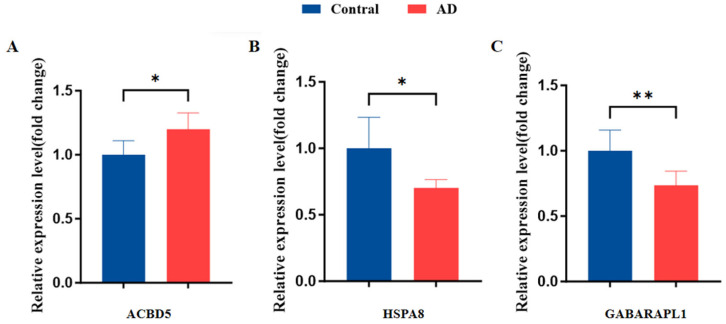
Validation of the three hub genes expression via qRT-PCR in mice (n = 6 in the AD and control group). (**A**) *ACBD5* expression. (**B**) *HSPA8* expression. (**C**) *GABARAPL1* expression. (* *p* < 0.05, ** *p* < 0.01).

The differential expression of the hub genes identified in the bioinformatics analysis was validated by qRT-PCR using brain tissue samples from the AD mice and control mice. In line with the computational findings, the expression of *ACBD5* was significantly elevated in the AD mice compared to the controls, whereas the expression of *HSPA8* and *GABARAPL1* was reduced in the AD mice (Figure 11).

## 4. Discussion

Alzheimer’s disease (AD) is a neurodegenerative disorder influenced by a combination of genetic, environmental, and lifestyle factors. Despite extensive research efforts, the precise etiology of AD remains elusive, presenting substantial challenges for diagnosis and treatment. Recent evidence suggests that disruptions in lipid metabolism [28] and autophagy [14] play significant roles in the pathogenesis of AD. Furthermore, the application of bioinformatics techniques [29] shows considerable promise in elucidating the complex mechanisms underlying the disease, offering valuable insights into potential diagnostic markers and therapeutic targets.

In this study, we conducted a comprehensive analysis of transcriptomic data from the GEO database to identify potential biomarkers associated with lipid metabolism and autophagy in AD. Utilizing WGCNA and machine learning techniques, we identified three hub genes—*ACBD5*, *GABARAPL1*, and *HSPA8*—that are critically involved in AD pathology. Based on these hub genes, we developed a diagnostic nomogram that demonstrated excellent predictive performance and clinical utility in both training and validation datasets. Additionally, we performed analyses of consensus clustering, immune cell infiltration, regulatory networks, functional enrichment, and drug prediction to further unravel the complex mechanisms and potential therapeutic targets related to these biomarkers in AD.

The *ACBD5* gene encodes a protein known as acyl-coenzyme A binding domain-containing protein 5, which plays a crucial role in various cellular metabolic processes. Specifically, it is involved in the sequestration, transport, and distribution of long-chain acyl-coenzyme A molecules [30]. Research indicates that mutations in the *ACBD5* gene can lead to the accumulation of very long-chain fatty acids (VLCFA), which result in central nervous system (CNS) lesions, including severe leukodystrophy, retinal dystrophy, ataxia, and psychomotor delay [31]. While there has been limited research directly examining the relationship between *ACBD5* and AD, our study contributes to this field by demonstrating that *ACBD5* may possess diagnostic value, evidenced by an AUC value exceeding 0.80. As an integral peroxisomal membrane protein, *ACBD5* was shown to facilitate lipid exchange between the endoplasmic reticulum (ER) and peroxisomes, which is critical for lipid synthesis, catabolism, and cholesterol trafficking in the brain [32,33]. Peroxisomal alterations have been implicated in the pathogenesis of Alzheimer’s disease, as evidenced by findings such as increased VLCFAs and decreased plasmalogen levels in the brains of advanced AD patients. These changes may disrupt peroxisomal lipid metabolism, potentially contributing to AD progression. Furthermore, defects in organelle transport, particularly the absence of peroxisome volume in processes linked to phosphorylated tau, indicate compromised transport during early neuronal degeneration [34]. Initial studies indicate that targeting peroxisomes could mitigate cognitive impairment and neurodegeneration in AD models, highlighting the potential of *ACBD5* as a therapeutic target for Alzheimer’s disease.

GABA type A receptor-associated protein-like 1 (*GABARAPL1*) is a member of the GABARAP protein family, which plays a crucial role in various cellular processes, including autophagy and receptor transportation to the plasma membrane, thereby maintaining cellular homeostasis [35]. Disruption of *GABARAPL1* function has been linked to neuroinflammation in the CNS [36]. Our study aligns with previous research that has reported alterations in *GABARAPL1* expression in in vitro models relevant to AD [37], as well as in cellular models of AD and type 2 diabetes mellitus comorbidity [38]. *GABARAPL1* is known to associate with autophagosomes or lysosomes, playing a critical role in the autophagy process [39]. Upon activation of autophagy, *GABARAPL1* becomes lipidated on the autophagosome membrane, facilitating subsequent autophagic events. This lysosome-based process is essential for recycling cellular components, eliminating damaged organelles, and degrading aggregation-prone proteins. Autophagy–lysosomal dysfunction has been well-documented in Alzheimer’s disease, contributing to the accumulation of amyloid-beta and tau proteins [40]. Targeting *GABARAPL1* may restore autophagic function, enhance the degradation of pathological proteins, and improve cognitive outcomes in AD.

HSPA8/HSC70, a member of the heat shock protein (HSP) family, is integral to various cellular processes, such as regulating cell division, transcriptional and translational control, chaperone activity, and signaling [41]. Notably, previous studies have reported the accumulation of HSPA8/HSC70 protein (Hsc70) in Parkinson’s disease models, indicating its potential involvement in neurodegenerative disorders [42]. Additionally, prior research has demonstrated that overexpression of *HSPA8* significantly reduces the accumulation of brain Aβ plaques and reverses molecular and behavioral phenotypes associated with AD [43]. Our findings align with this literature, as we also observe a significant association between *HSPA8* and AD. Mechanistically, the molecular chaperone Hsc70 (*HSPA8*) plays a crucial role in chaperone-mediated autophagy (CMA), a key pathway for eliminating AD-related pathological proteins [44]. Research has demonstrated a decline in the metabolic activity of lipid droplets in AD models, which contributes to increased oxidative stress and inflammation, thereby exacerbating neurotoxic effects. The CMA pathway is known to initiate lipolysis and lipophagy by degrading lipid droplet-associated proteins, including perilipin 2 (PLIN2) and perilipin 3 (PLIN3) [45]. In light of these findings, our study posits that *HSPA8* is significantly involved in AD through its regulatory functions in autophagy and lipid homeostasis. This highlights the potential therapeutic implications of targeting *HSPA8* to enhance autophagic processes and restore lipid balance, offering a promising avenue for intervention in Alzheimer’s disease.

Lipids are essential for maintaining neuronal integrity, synaptic function, and cellular signaling in the brain. Recent evidence suggests that AD may be a metabolic disorder, with numerous studies linking lipid metabolism dysfunction to AD. Dysregulation of lipid metabolism, including alterations in cholesterol, fatty acids, and phospholipids, has been implicated in the development of AD by affecting the synthesis and clearance of neurotoxic amyloid-beta (Aβ) protein [28]. Autophagy, a key cellular pathway for degrading damaged proteins and organelles, also plays a critical role in AD. Impaired autophagy leads to the accumulation of toxic protein aggregates, such as Aβ and tau, which are hallmarks of the disease. There is a significant interplay between autophagy and lipid metabolism in AD; deficiencies in autophagy can disrupt microglial lipid homeostasis, worsening tau pathology [16], while accumulated LDs can lead to lysosomal dysfunction, further impairing autophagy [46]. Moreover, lipophagy, a form of lipid degradation, has emerged as a crucial factor in AD pathogenesis. By employing bioinformatics techniques, we have identified several biomarkers associated with lipid metabolism and autophagy, offering insights into the molecular mechanisms underlying Alzheimer’s disease.

Neuroinflammation plays a critical role in the pathology of Alzheimer’s disease, with immune cells significantly contributing to this process. Evidence suggests that risk genes associated with AD are linked to immune functions, indicating that both innate and adaptive immunity are activated in the context of the disease [47]. In our study, immune cell infiltration allowed us to categorize AD samples into two distinct clusters, A and B, using unsupervised clustering based on the expression of *ACBD5*, *GABARAPL1*, and *HSPA8*. Notably, we observed significant diversity in immune patterns between these two subclusters. T cells, including CD8 T cells, CD4 T cells, T helper cells, and Tregs, were found to have a strong correlation with the three hub genes. This finding aligns with previous research indicating that T-cell dysfunction is a crucial modulator of AD pathogenesis [48]. Aberrant T cells may indirectly contribute to AD by inducing neuroinflammation through the secretion of pro-inflammatory mediators and the activation of immune responses. Additionally, other immune cells, such as natural killer cells, dendritic cells, and monocytes, exhibited alterations in cerebrospinal fluid and peripheral blood from AD patients [49], although the underlying mechanisms remain unclear. These findings suggest that the three hub genes may influence AD development by modulating the immune microenvironment.

The GSEA results indicated that the hub genes *ACBD5*, *GABARAPL1*, and *HSPA8* are involved in several critical biological processes, including the proteasome, oxidative phosphorylation, the TCA cycle, and aminoacyl-tRNA biosynthesis. The proteasome is essential for regulating protein turnover, maintaining quality control, and eliminating oxidized or misfolded proteins. Previous studies have suggested that proteasome inhibition may contribute to synaptic and memory deficits in AD by inducing reactive oxygen species, causing dendritic spine loss, and inhibiting mRNA translation in the hippocampus [50]. Recent evidence has identified mitochondrial dysfunction as a key mediator of AD pathogenesis. Impairments in oxidative phosphorylation and the TCA cycle can lead to mitochondrial malfunction, resulting in energy metabolism deficiencies and free radical damage [51]. Consistent with our findings, proteomic analyses have previously confirmed disruptions in aminoacyl-tRNA biosynthesis in neuroglioma cells carrying the *ApoE4* allele [52]. These findings underscore the involvement of *ACBD5*, *GABARAPL1*, and *HSPA8* in AD progression through their participation in various biological pathways.

We identified several regulatory components, including transcription factors and microRNAs (miRNAs), and predicted various chemical compounds as potential therapeutic agents. Among these, TRIM28 emerged as a key transcription factor that regulates the expression of *HSPA8* and *GABARAPL1*. As a prominent member of the TRIM (tripartite motif) family, TRIM28 possesses essential functional domains for transcriptional regulation, primarily through interactions with KRAB-ZNF proteins. Previous studies have indicated that TRIM28 modulates tau protein levels, with reduced adult TRIM28 leading to decreased tau expression [53]. miRNAs also play a significant role in post-transcriptional gene regulation. An in vivo study demonstrated that hsa-miR-16-5p is associated with altered protein levels in the hippocampus of individuals with AD [54]. This study further revealed that hsa-miR-16-5p regulates all three identified hub genes. Additionally, several chemical compounds were recognized as potential drugs. Notably, parthenolide has been shown to alleviate cognitive dysfunction and neurotoxicity by modulating the AMPK/GSK3β(Ser9)/Nrf2 signaling pathway in AD [55]. These identified regulatory components not only serve as valuable biomarkers but also hold promise for therapeutic applications.

This study has several limitations. First, we validated the hub genes exclusively in P301S mice, which may limit the applicability of our findings. To enhance the robustness of our conclusions, further validation in additional Alzheimer’s models, such as APPPS1 mice, is essential, as it would allow for a more comprehensive understanding of the underlying pathological mechanisms in Alzheimer’s disease. While the identification of *ACBD5* as a novel target gene is significant, the prior validation of *GABARAPL1* and *HSPA8* underscores the need for caution in interpreting their roles within our context. Future research should aim to elucidate the interactions of these proteins in lipid metabolism and their potential implications for Alzheimer’s disease, thereby enhancing our understanding of their functions in neurodegeneration. Additionally, while the nomogram demonstrates strong performance in publicly available datasets, the absence of independent clinical validation significantly restricts its translational impact and applicability in real-world settings. Therefore, it is imperative for future studies to undertake independent validations to affirm the nomogram’s efficacy in clinical practice.

## 5. Conclusions

In conclusion, our study employed WGCNA and three machine learning algorithms to identify three hub genes and construct a predictive nomogram, which were subsequently validated through public databases and animal experiments. These findings have the potential to reveal new targets and advance the development of innovative diagnostic and therapeutic strategies for Alzheimer’s disease.

## Figures and Tables

**Table 1:** Potential drugs of LA/Lipo-related hub genes.

Term	Adjusted *p*-Value	Odds Ratio	Combined Score	Genes
1,4-chrysenequinone	0.004290494	886.7555556	9781.874312	*HSPA8*; *GABARAPL1*
thiostrepton	0.014931296	305.6461538	2736.968135	*HSPA8*; *GABARAPL1*
parthenolide	0.014931296	266.4161074	2313.934025	*HSPA8*; *GABARAPL1*
15-delta prostaglandin J2	0.016810729	216.5464481	1792.825598	*HSPA8*; *GABARAPL1*
puromycin	0.029464858	145.0367647	1087.028637	*HSPA8*; *GABARAPL1*
menadione	0.03643546	118.4638554	841.1150706	*HSPA8*; *GABARAPL1*

## Data Availability

These data were derived from the Gene Expression Omnibus (GEO) database (https://www.ncbi.nlm.nih.gov/geo/, accessed on 18 December 2023). Further inquiries can be directed to the corresponding author.

## Supplementary Materials

The following supporting information can be downloaded at: https://www.mdpi.com/article/10.3390/biomedicines13020362/s1, Table S1. The detailed lipid metabolism, autophagy, and lipophagy-related gene sets from the GSEA and HADb database; Table S2. Primer sequences of hub genes.
