# Supplementary material for: Identification of Lipophagy-Related Gene Signature for Diagnosis and Risk Prediction of Alzheimer’s Disease
Source: Biomedicines. 2025 Feb 5;13(2):362. doi: 10.3390/biomedicines13020362 (PMC11853564; doi:10.3390/biomedicines13020362)
Supplement: Supplementary file 1 [file biomedicines-13-00362-s001.zip › biomedicines-3393834-supplementary.pdf]

Identification of Lipophagy-Related Gene Signature for Diagnosis and Risk Prediction of Alzheimer's Disease

Table S1. The detailed lipid metabolism, autophagy, and lipophagy-related gene sets from the GSEA and HADb database.

**Database**	**Gene Set**	**Gene Count**
	lipid metabolism-related genes	
GSEA	GOBP_GLYCEROLIPID_METABOLIC_PROCESS	390
	GOBP_PHOSPHOLIPID_METABOLIC_PROCESS	376
	GOBP_MEMBRANE_LIPID_METABOLIC_PROCESS	210
	HALLMARK_FATTY_ACID_METABOLISM	158
	KEGG_FATTY_ACID_METABOLISM	42
	KEGG_GLYCEROLIPID_METABOLISM	49
	KEGG_GLYCEROPHOSPHOLIPID_METABOLISM	77
	KEGG_SPHINGOLIPID_METABOLISM	40
	KEGG_ETHER_LIPID_METABOLISM	33
	REACTOME_ARACHIDONIC_ACID_METABOLISM	62
	REACTOME_FATTY_ACID_METABOLISM	195
	REACTOME_GLYCOSPHINGOLIPID_METABOLISM	57
	REACTOME_METABOLISM_OF_LIPIDS	835
	REACTOME_PHOSPHOLIPID_METABOLISM	248
	REACTOME_SPHINGOLIPID_METABOLISM	103
	REACTOME_TRIGLYCERIDE_METABOLISM	40
	WP_CHOLESTEROL_METABOLISM	72
	WP_GLYCOSPHINGOLIPID_METABOLISM	21
	WP_LIPID_METABOLISM_PATHWAY	29
	WP_SPHINGOLIPID_METABOLISM_INTEGRATED_PATHWAY	26
Sum		3063(unique:1190)
	**Autophagy-related genes**	
GSEA		
	GOBP_CHAPERONE_MEDIATED_AUTOPHAGY	15
	GOBP_LIPOPHAGY	7
	GOBP_MITOPHAGY	39
	GOBP_POSITIVE_REGULATION_OF_AUTOPHAGY	150
	GOBP_REGULATION_OF_AUTOPHAGY	358
	GOBP_SELECTIVE_AUTOPHAGY	97
	KEGG_REGULATION_OF_AUTOPHAGY	35
	REACTOME_AUTOPHAGY	163
	REACTOME_SELECTIVE_AUTOPHAGY	91
	WP_AUTOPHAGY	30

Database	Gene Set	Gene Count
	GOCC_AUTOLYSOSOME	12
	GOBP_REGULATION_OF_AUTOPHAGY_OF_MITOCHONDRION_IN_RESPONSE_TO_MITOCHONDRIAL_DEPOLARIZATION	15
	GOBP_PEXOPHAGY	6
	GOBP_NEGATIVE_REGULATION_OF_AUTOPHAGY	92
HADb		222
Sum		1332(unique:661)
	**lipophagy-related genes**	
GSEA	REACTOME_LIPOPHAGY	9
Sum		9

Table S2. Primer sequences of hub genes

Primer name	Sequences (5'to 3')
ACBD5_F	ATCTTCCGGGTGGGAGAAAG
ACBD5_R	TCATACACTGACGGTGTGTCC
GABARAPL1_F	AGGACCACCCCTTCGAGTATC
GABARAPL1_R	GCACAAGGTACTTCCTCTTATCC
HSPA8_F	TCTCGGCACCACCTACTCC
HSPA8_R	CTACGCCCGATCAGACGTTT
ACTB_F	GGCTGTATTCCCCTCCATCG
ACTB_R	CCAGTTGGTAACAATGCCATGT
